# Association of lncRNA H19 rs217727 polymorphism and cancer risk in the Chinese population: a meta-analysis

**DOI:** 10.18632/oncotarget.10936

**Published:** 2016-07-29

**Authors:** Yanjun Lu, Lu Tan, Na Shen, Jing Peng, Chunyu Wang, Yaowu Zhu, Xiong Wang

**Affiliations:** ^1^ Department of Laboratory Medicine, Tongji Hospital, Tongji Medical College, Huazhong University of Science and Technology, Wuhan 430030, China; ^2^ Key Laboratory for Molecular Diagnosis of Hubei Province, The Central Hospital of Wuhan, Tongji Medical College, Huazhong University of Science and Technology, Wuhan, Hubei 430014, China

**Keywords:** IncRNA, rs217727, polymorphism, cancer, meta-analysis

## Abstract

Reports on the relationship between the lncRNA H19 rs217727 polymorphism and the risk of cancer in the Chinese population have been inconsistent. Therefore, we performed a meta-analysis to evaluate this association, by searching the Embase, PubMed, Web of Science, Wanfang, and CNKI databases. Four case-control studies with 3,157 cases and 3,564 controls were selected for this meta-analysis. The odds ratios with 95% confidence intervals were examined using the random effect model. Allelic (A vs. G), dominant (AA + GA vs. GG), recessive (AA vs. GA + GG), and additive (AA vs. GG) genetic models were used to determine the association. Overall, no significant association was observed between the rs217727 polymorphism and cancer susceptibility in any of the four genetic models. Sensitivity analysis revealed that the results were stable in the allelic and dominant genetic models, but those from the recessive and additive models were unstable, which should be treated with caution. Our meta-analysis suggests that the lncRNA H19 rs217727 polymorphism might not be associated with overall cancer risk. However, well-designed, large-scale studies with different ethnic populations need to be conducted in the future to elucidate the potential association.

## INTRODUCTION

Various long non-coding RNAs (lncRNAs) have been known to play a role in various diseases, including cancer, via transcriptional and post-transcriptional regulation of the expression of oncogenes or tumor suppressors [1]. The lncRNA H19, located on human chromosome 11p15.5, belongs to the lncRNA family, which comprises mRNA-like transcripts lacking an open reading frame. LncRNA H19 is a maternal expressed gene that plays essential roles in embryogenesis and whose expression is decreased in maturing tissues [2]. Several studies suggest that aberrant lncRNA H19 expression may contribute to the carcinogenesis of many cancers. LncRNA H19 has been found to be aberrantly expressed in many cancers including breast, esophageal, bladder, and colorectal cancers and may be involved in the carcinogenesis of these cancers by targeting the downstream microRNAs [3–6]. In addition, progressive up-regulation of lncRNA H19 was found in advanced stages of gastric cancer, and circulating lncRNA H19 has been suggested as a potential novel biomarker in gastric cancer [7]. Moreover, upregulation of lncRNA H19 indicates poor prognosis in gallbladder carcinoma [8]. A recent meta-analysis by Chen et al. also showed that high levels of lncRNA H19 could predict poor overall survival and lymph node metastasis in multiple cancers [9]. Some other molecules including methyl-CpG binding protein 2 (MeCP2), c-Myc, and Yes-associated protein 1 (YAP1) have also been found to modulate the expression of lncRNA H19 [10–12]. In addition to these molecules, single nucleotide polymorphisms (SNPs) within lncRNA H19 have been found to regulate its expression and function as well [13, 14]. The lncRNA H19 rs217727 polymorphism has been shown to be associated with susceptibility to gastric cancer, breast cancer, and bladder cancer in the Chinese population [5, 14, 15]. However, the results in different cancers have been inconsistent. For example, Verhaegh et al. showed that the rs217727 polymorphism might not be associated with bladder cancer in a Caucasian population even if the subjects are grouped by tumor stage or grade [13]. In contrast, Hua et al. showed increased bladder cancer risk in the recessive genetic model; their stratified analyses in subgroups of young subjects, males, smokers, and high tumor grade also revealed similar results [5]. In contrast, Li et al. found that the rs217727 polymorphism was not associated with colorectal cancer [6], and Xia et al. found that the rs217727 polymorphism was associated with breast cancer risk only in the dominant genetic model [15].

Therefore, to achieve a more accurate evaluation of the association between the lncRNA H19 rs217727 polymorphism and overall cancer risk in the Chinese population, we performed this meta-analysis with an aim to explore the role of rs217727 in cancer carcinogenesis and to explain the possible reasons for the inconsistent results reported so far in previous studies.

## RESULTS

### Characteristics of the published studies

**Figure 1:**
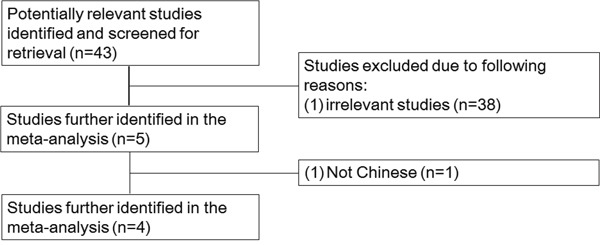
Flow diagram of literature search and selection

The study selection process was as shown in Figure 1. In total, 43 studies were selected using the search strategy. Of these, 38 irrelevant studies were excluded and five were screened further. Of the five studies, one was excluded because the authors had chosen a Caucasian population (Supplementary Table S1). Finally, four studies with 3,157 cases and 3,564 controls were included in the meta-analysis. The main characteristics of the selected four studies and the genotype distribution of the lncRNA H19 rs217727 polymorphism are summarized in Tables 1 and 2.

**Table 1:** Characteristics of 4 studies included in this meta-analysis

Author	Year	Method	Cancer type	Age		Sample size		Score
				Case	Control	Case	Control	
Hua Q	2016	TaqMan	bladder cancer	64.8±12.6	65.2±9.3	1046	1394	12
Li S	2016	TaqMan	colorectal cancer	60.0±12.6	59.9±14.3	1147	1203	14
Xia Z	2016	RFLP	breast cancer	48.4±10.3	48.9±10.0	464	467	13
Yang C	2015	TaqMan	gastric cancer	58.7±10.7	59.2±13.5	500	500	12

**Table 2:** Genotype frequencies of rs217727 in 4 studies included in this meta-analysis

Author	Year	Case			Control			MAF		HWE
		AA	GA	GG	AA	GA	GG	Case	Control	
Hua Q	2016	148	467	431	156	665	573	0.365	0.350	0.074
Li S	2016	153	514	480	177	570	456	0.357	0.384	0.959
Xia Z	2016	148	156	160	116	212	139	0.487	0.475	0.052
Yang C	2015	88	252	160	63	244	193	0.428	0.370	0.296

### Meta-analysis for rs217727

**Figure 2:**
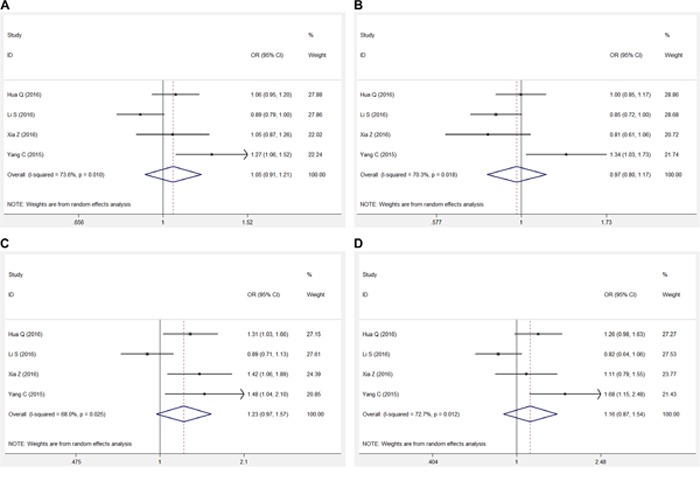
Forest plots for meta-analysis of rs217727 polymorphism and cancer risk in Chinese population **A.** Allelic model (A vs. G). **B.** Dominant genetic model (AA + GA vs. GG). **C.** Recessive genetic model (AA vs. GA + GG). **D.** Addictive genetic model (AA vs. GA).

The pooled results for the association between the lncRNA H19 rs217727 polymorphism and cancer risk are shown in Figure 2 and Table 3. In the overall analysis, the lncRNA H19 rs217727 polymorphism was not found to be significantly associated with cancer risk in the allelic (A vs. G), dominant (AA + GA vs. GG), recessive (AA vs. GA + GG), or additive (AA vs. GG) genetic models [A vs. G, odds ratio (OR): 1.051, 95% confidence interval (CI): 0.913, 1.211, *P* = 0.490; AA + GA vs. GG, OR: 0.970, 95% CI: 0.802, 1.174, *P* = 0.755; AA vs. GA + GG, OR: 1.232; 95% CI: 0.968, 1.568, *P* = 0.090; AA vs. GG, OR: 1.156, 95% CI: 0.868, 1.541, *P* = 0.321]. Stratified analysis based on the genotyping method also showed similar results in the Taqman-method subgroup (Table 3).

**Table 3:** Meta-analysis of rs217727 polymorphism and cancer risk in Chinese population

Genetic model	*P*_Q_	I^2^	OR	95% CI	*P*_Z_^*^
Overall					
A vs. G	0.010	73.6%	1.051	0.913, 1.211	0.490
AA + GA vs. GG	0.018	70.3%	0.970	0.802, 1.174	0.755
AA vs. GA + GG	0.025	68.0%	1.232	0.968, 1.568	0.090
AA vs. GG	0.012	72.7%	1.156	0.868, 1.541	0.321
Method (Taqman)					
A vs. G	0.004	82.3%	1.055	0.876, 1.271	0.572
AA + GA vs. GG	0.015	76.2%	1.021	0.814, 1.280	0.859
AA vs. GA + GG	0.022	73.8%	1.181	0.871, 1.601	0.285
AA vs. GG	0.004	81.8%	1.181	0.796, 1.752	0.409

* Random effect model was used.

A meta-regression was performed to identify the source of the heterogeneity by considering the publication year, MAF of cases (> 0.4 vs. ≤ 0.4), sample size (> 2000 vs. ≤ 2000), and genotyping method (Taqman vs. RFLP) as possible covariates; however, the meta-regression analysis did not reveal any covariate significantly contributing to the heterogeneity in any genetic model (data not shown).

### Sensitivity analysis

Sensitivity analysis showed that the pooled ORs were not materially influenced by any single study in the allelic and dominant genetic models, indicating that our results were statistically robust in the allelic and dominant genetic models (Table 4). In the recessive and additive genetic models, after omitting the study by Li et al., rs217727 was found to be significantly associated with cancer risk (recessive, OR: 1.379, 95% CI: 1.172, 1.624, *P* < 0.001; additive, OR: 1.301, 95% CI: 1.053, 1.608, *P* = 0.015). These data suggest that our meta-analysis results were not stable in the recessive and additive genetic models, but remained stable in the allelic and dominant genetic models.

**Table 4:** Sensitivity analysis of the meta-analysis

Genetic model	*P*_Q_	I^2^	OR	95% CI	*P*_Z_
A vs. G					
Hua Q	0.005	81.5%	1.052	0.850, 1.301	0.643
Li S	0.209	36.2%	1.114	0.995, 1.247	0.062
Xia Z	0.004	82.3%	1.055	0.876, 1.271	0.572
Yang C	0.092	58.0%	0.993	0.879, 1.121	0.904
AA + GA vs. GG					
Hua Q	0.008	79.3%	0.964	0.719, 1.294	0.809
Li S	0.030	71.6%	1.024	0.798, 1.314	0.852
Xia Z	0.015	76.2%	1.021	0.814, 1.280	0.859
Yang C	0.272	23.1%	0.898	0.793, 1.018	0.092
AA vs. GA + GG					
Hua Q	0.014	76.7%	1.214	0.861, 1.711	0.270
Li S	0.826	0.0%	1.379	1.172, 1.624	*P*<0.001
Xia Z	0.022	73.8%	1.181	0.871, 1.601	0.285
Yang C	0.021	74.0%	1.174	0.882, 1.563	0.271
AA vs. GG					
Hua Q	0.008	79.1%	1.130	0.754, 1.692	0.554
Li S	0.263	25.1%	1.301	1.053, 1.608	0.015
Xia Z	0.004	81.8%	1.181	0.796, 1.752	0.409
Yang C	0.058	64.8%	1.042	0.795, 1.366	0.765

### Publication bias

Begg's and Egger's tests were performed to assess the publication bias of the studies, and no significant publication bias was found (Table 5), which indicated that our meta-analysis results are reliable.

**Table 5:** Publication bias analysis of the meta-analysis

Genetic model	Test	t	95% CI	*P*
A vs. G	Begg's test			1.000
	Egger's test	1.18	−14.020, 24.609	0.360
AA + GA vs. GG	Begg's test			1.000
	Egger's test	0.43	−17.800, 21.745	0.710
AA vs. GA + GG	Begg's test			0.308
	Egger's test	1.33	−15.359, 29.044	0.316
AA vs. GG	Begg's test			0.308
	Egger's test	1.28	−15.351, 28.315	0.330

## DISCUSSION

LncRNA H19, as an imprinted gene, servers as an oncogene and was aberrantly increased in several cancers, and it might promote carcinogenesis via acting as competitive endogenous RNAs (ceRNA) or precursors of microRNAs. Liu L et al., reported that lncRNA H19 promoted thyroid carcinogenesis by competitively binding miR-17-5p to regulate its target YES1 expression, functioning as ceRNA [16]. LncRNA H19 has been shown to function as precursor of miR-675 and act as an oncogene in a variety of cancers, including bladder cancer, gastric cancer, glioma, and colorectal cancer [17–20]. The differentially methylated regions (DMRs), located upstream of H19, could modulate H19 gene expression [21]. Furthermore, genetic variants of lncRNA H19 have been shown to affect its expression and susceptibility to cancers [13, 14]. Previous studies have shown inconsistent results with regard to the association between the lncRNA H19 rs217727 polymorphism and cancer risk, which might be because the studies focused on different cancer types and different ethnic populations or because of inadequate statistical power [5, 13]. Thus, we carried out this meta-analysis with an aim to acquire an accurate evaluation of this association.

**Figure 3:**
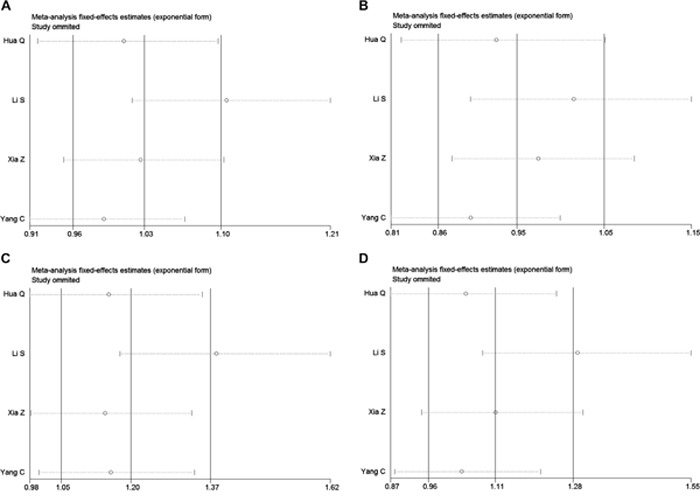
Sensitivity analysis for meta-analysis of rs217727 polymorphism and cancer risk in Chinese population **A.** Allelic model (A vs. G). **B.** Dominant genetic model (AA + GA vs. GG). **C.** Recessive genetic model (AA vs. GA + GG). **D.** Addictive genetic model (AA vs. GA).

In the current meta-analysis, we comprehensively analyzed all literatures studying the association between the lncRNA H19 rs217727 polymorphism and cancer risk in the Chinese population; however, we did not find any significant association in any genetic model in the overall Chinese population and in our stratification analysis based on the genotyping method (Figure 2, Table 3). Meta-regression was then used to investigate the source of heterogeneity by considering possible covariates like publication year, MAF of cases, sample size, and genotyping method, but no covariate significantly contributed to the heterogeneity. Sensitivity analysis showed that our results were statistically robust in the allelic and dominant genetic models, but not in the recessive and additive genetic models and these results should be treated with caution (Figure 3). No significant publication bias was found in our meta-analysis.

Different cancer types might contribute to the overall result of our meta-analysis. In our meta-analysis, bladder cancer, colorectal cancer, breast cancer, and gastric cancer were included, but each type of cancer only included one study, thus making stratification impossible. Furthermore, different cancers may also be affected by gender, smoking habits, and other subgroup populations, suggesting that studies on a larger scale including different cancers need to be conducted in the future. Different sex ratios may also affect the overall result In the study on breast cancer by Xia et al., due to the special characteristics, only women were included [15]. In the studies conducted by Li and Yang et al., the sex ratios (male/female) were around 0.5, while in Hua et al.'s study, this ratio was around 0.8, although the sex ratio was matched between the case and control groups [5]. In addition to the sex ration, age may also affect the overall result. The mean age of the study subjects in Xia et al.'s study was around 50 years, while that in the other three studies was around 60 years. Some other factors including smoking habit and cancer stage may also contribute to the overall result of meta-analyses. For example, as mentioned previously, Hua et al. showed that the rs217727 polymorphism was associated with bladder cancer risk in smokers, while Yang et al. showed an association with gastric cancer in non-smokers [5, 14]. These factors may have a complex contribution to the overall heterogeneity and the result of meta-analysis. Therefore, studies with larger sample sizes and rigorous designs are needed to clarify this association.

Our current meta-analysis had some limitations that will need to be addressed in future studies. First, only the Chinese population was analyzed, as only one study with a Caucasian population was found. We also performed an overall meta-analysis including both Caucasian and Chinese populations, and the result was similar to that found when only the Chinese population was considered (Supplementary Table S2). Second, we did not perform stratification by cancer type, cancer stage, sex, and smoking habit, owing to limited literature and unavailability of detailed allele frequency data: Hua et al.'s study provided stratified analysis only in the recessive genetic model, Li et al. did not provide stratified analysis of this variant, while Xia et al. and Yang et al. performed stratified analysis only in the dominant genetic model. Third, the sample size was small.

Nevertheless, our study showed that the lncRNA H19 rs217727 polymorphism might not be associated with cancer rick in the Chinese population. However, further case-control studies with larger sample sizes in different ethnic populations will be needed to achieve more definitive results.

## MATERIALS AND METHODS

### Literature search

Eligible studies were systematically searched in Embase, PubMed, Web of Science, Wanfang, and CNKI databases up to July 6, 2016, with keywords including “long Noncoding RNA H19 OR lncRNA H19 OR long non-coding RNA H19” and “polymorphism OR variation OR rs217727” and “cancer OR carcinoma OR tumor”. We also manually examined reference lists for relevant publications.

### Inclusion and exclusion criteria

Eligible studies should meet the following criteria: a) studies evaluating the association between lncRNA H19 rs217727 polymorphism and the risk of cancer in Chinese population, b) case-control designed studies, c) studies with sufficient genotype data for calculation of the odds ratio (OR) with 95% confidence intervals (95% CIs). Exclusion criteria were as follows: a) reviews, b) studies lacked detailed genotype information, c) studies included overlapping subjects, d) studies on other population rather than Chinese population, e) studies not on human, f) studies not on lncRNA H19 rs217727. In cases where overlapping subjects were included, the study with the larger sample size was selected.

### Data extraction

Two investigators (Yanjun Lu, Lu Tan) independently extracted the following data from each article with a standardized protocol: first author name, publication year, Country, ethnicity, age, genotyping method, sample size, genotype, allele frequency, source of control, minor allele frequence (MAF), *P* value for Hardy-Weinberg equilibrium (HWE). Disparities, if any, were resolved via discussion.

### Quality score

The quality of the studies was independently evaluated by two reviewers (Na Shen, Jing Peng) according to quality assessment scale (Table 6). Total scores ranged from 0 (worst) to 15 (best) [22].

**Table 6:** Quality score assessment

Criterion Score	Score	Hua Q	Li S	Xia Z	Yang C
**Source of cases**					
Selected from population or cancer registry	3				
Selected from hospital	2	2	2	2	2
Selected from pathology archives, but without description	1				
Not described	0				
**Source of controls**					
Population-based	3		3	3	
Blood donors or volunteers	2				
Hospital-based (cancer-free patients)	1	1			1
Not described	0				
**Specimens used for determining genotypes**					
White blood cells or normal tissues	3	3	3	3	3
Tumor tissues or exfoliated cells of tissue	0				
**Hardy–Weinberg equilibrium in controls**					
Hardy–Weinberg equilibrium	3	3	3	3	3
Hardy–Weinberg disequilibrium	0				
**Total sample size**					
≥1,000	3	3	3		3
≥500 and <1,000	2			2	
≥200 and <500	1				
<200	0				
**Total score**		12	14	13	12

### Statistical analysis

The crude ORs and 95% CIs were calculated to measure the association between lncRNA H19 rs217727 polymorphism and cancer risk. Four genetic models were used: allelic (A vs. G), dominant (AA + GA vs. GG), recessive (AA vs. GA + GG), and additive (AA vs. GG) genetic models. HWE was examined by χ^2^ test in the controls. Cochran Q test and I^2^ statistic were used to assess heterogeneity. If *P* value for Q test>0.1 or I^2^<50%, fixed effect model was used; otherwise, random effect model was chosen. The significance of the pooled OR was determined with Z test, and *P*<0.05 was considered to be statistically significant. Sensitivity analysis was conducted by sequentially omiting one single study at a time and recounting ORs and 95% CIs. Egger's and Begg's tests were applied to detect the potential publication bias. Meta regression analysis was performed to estimate the main source of heterogeneity. All statistical tests were analyzed by STATA 11.0 (STATA Corporation, College Station, TX, USA).

## SUPPLEMENTARY TABLES




