# Supplementary material for: Association of lncRNA H19 rs217727 polymorphism and cancer risk in the Chinese population: a meta-analysis
Source: Oncotarget. 2016 Jul 29;7(37):59580–8. doi: 10.18632/oncotarget.10936 (PMC5312333; doi:10.18632/oncotarget.10936)
Supplement: Supplementary file 1 [file oncotarget-07-59580-s001.pdf]

Association of lncRNA H19 rs217727 polymorphism and cancer risk in the Chinese population: a meta-analysis

SUPPLEMENTARY TABLES

Supplementary Table S1: Excluded studies and exclusion criteria

See Supplementary File 1

Supplementary Table S2: Meta-analysis of rs217727 polymorphism and cancer risk

Genetic model	P_Q	I^2	OR	95% CI	P_Z*
A vs. G	0.006	72.6%	1.013	0.879, 1.168	0.856
AA + GA vs. GG	0.018	66.3%	0.936	0.782, 1.120	0.467
AA vs. GA + GG	0.023	64.9%	1.192	0.931, 1.526	0.164
AA vs. GG	0.011	69.5%	1.105	0.826, 1.479	0.502

*Random effect model was used.
