# Supplementary material for: Association of lncRNA H19 rs217727 polymorphism and cancer risk in the Chinese population: a meta-analysis
Source: Oncotarget. 2016 Jul 29;7(37):59580–8. doi: 10.18632/oncotarget.10936 (PMC5312333; doi:10.18632/oncotarget.10936)
Supplement: Supplementary file 2 [file oncotarget-07-59580-s002.docx]

Table S1 Excluded studies and exclusion criteria

| Title | Exclusion criteria |
| --- | --- |
| 1. Association of well-characterized lung cancer lncRNA polymorphisms with lung cancer susceptibility and platinum-based chemotherapy response | Other varitant of lncRNA H19, not rs217727 |
| 1. A Common Polymorphism within the IGF2 Imprinting Control Region Is Associated with Parent of Origin Specific Effects in Infantile Hemangiomas | Polymorphism of IGF-II, not lncRNA H19 |
| 1. H19 DMR methylation correlates to the progression of esophageal squamous cell carcinoma through IGF2 imprinting pathway | LncRNA H19 DMR, not rs217727 |
| 1. Determination of allelic expression of h19 in pre- and peri-implantation mouse embryos | Not human being |
| 1. Investigation of IGF2/ApaI and H19/RsaI polymorphisms in patients with cutaneous melanoma | Without control group |
| 1. Inter- and intra-individual variation in allele-specific DNA methylation and gene expression in children conceived using assisted reproductive technology | LncRNA H19 DMR, not rs217727 |
| 1. Polymorphisms in the H19 gene and the risk of bladder cancer | Caucasian, not Chinese |
| 1. A methyl-deficient diet modifies histone methylation and alters Igf2 and H19 repression in the prostate | Not human being |
| 1. Examination of IGF2 and H19 loss of imprinting in bladder cancer | LncRNA H19 DMR, not rs217727 |
| 1. Hepatocellular carcinomas of the albumin SV40 T-antigen transgenic rat display fetal-like re-expression of lgf2 and deregulation of H19 | Not human being |
| 1. Changes in DNA methylation patterns in subjects exposed to low-dose benzene | DNA methylation |
| 1. Genes harbouring susceptibility SNPs are differentially expressed in the breast cancer subtypes | Other varitant of lncRNA H19, not rs217727 |
| 1. The aberrant imprinting of insulin-like growth factor II and H19 in human hepatocellular carcinoma | Not rs217727 polymorphism |
| 1. Loss of imprinting of the insulin-like growth factor 2 and the H19 gene in testicular seminomas detected by real-time PCR approach | Not rs217727 polymorphism |
| 1. Genomic profiling maps loss of heterozygosity and defines the timing and stage dependence of epigenetic and genetic events in Wilms' tumors | Not rs217727 polymorphism |
| 1. Imprinting, expression, and localisation of DLK1 in Wilms tumours | DLK1, not lncRNA H19 |
| 1. A loss of insulin-like growth factor-2 imprinting is modulated by CCCTC-binding factor down-regulation at senescence in human epithelial cells | Cell culture, not human being |
| 1. Allele-specific methylation analysis on upstream promoter region of H19 by methylation-specific PCR with confronting two-pair primers | DNA methylation |
| 1. Biallelic expression of the H19 gene during spontaneous hepatocarcinogenesis in the albumin SV40 T antigen transgenic rat | Not human being |
| 1. Relaxation of imprinting of IGFII gene in juvenile nasopharyngeal angiofibromas | DNA methylation |
| 1. Altered imprinting, promoter usage, and expression of insulin-like growth factor-II gene in gestational trophoblastic diseases | Not rs217727 polymorphism |
| 1. Loss of imprinting in colorectal cancer linked to hypomethylation of H19 and IGF2 | DNA methylation |
| 1. Alterations in promoter usage and expression levels of insulin-like growth factor-II and H19 genes in cervical carcinoma exhibiting biallelic expression of IGF-II | Not rs217727 polymorphism |
| 1. Loss of imprinting and loss of heterozygosity on 11p15.5 in head and neck squamous cell carcinomas | Not rs217727 polymorphism |
| 1. Imprinting defects in mouse embryos: stochastic errors or polymorphic phenotype? | Not rs217727 polymorphism |
| 1. Imprinting of insulin-like growth factor 2 is modulated during hematopoiesis | Not rs217727 polymorphism |
| 1. p57(KIP2) is not mutated in hepatoblastoma but shows increased transcriptional activity in a comparative analysis of the three imprinted genes p57(KIP2), IGF2, and H19 | Not rs217727 polymorphism |
| 1. Genomic imprinting of IGF2 and H19 in human meningiomas | Not rs217727 polymorphism, no control group |
| 1. Loss of imprinting of the IGF-II and H19 genes in epithelial ovarian cancer | Not rs217727 polymorphism |
| 1. Disruption of imprinted genes at chromosome region 11p15.5 in paediatric rhabdomyosarcoma | Not rs217727 polymorphism |
| 1. Genomic imprinting of IGF-II and H19 in adult human pancreatic tissues | Not rs217727 polymorphism |
| 1. Genomic imprinting of H19 and insulin-like growth factor-2 in pediatric germ cell tumors | Not rs217727 polymorphism |
| 1. Proinsulin-like growth factor-II overexpression does not alter monoallelic H19 gene expression in transfected human embryonic kidney fibroblasts | Cell culture, not human being |
| 1. Frequent loss of imprinting of the H19 and IGF-II genes in ovarian tumors. | Not rs217727 polymorphism |
| 1. Loss of imprinting and overexpression of IGF2 gene in gastric adenocarcinoma | Not rs217727 polymorphism |
| 1. Coding mutations in p57KIP2 are present in some cases of Beckwith-Wiedemann syndrome but are rare or absent in Wilms tumors | Not rs217727 polymorphism |
| 1. Equivalent parental distribution of frequently lost alleles and biallelic expression of the H19 gene in human testicular germ cell tumors | Not rs217727 polymorphism |
| 1. Imprinting and expression of insulin-like growth factor-II and H19 in normal breast tissue and breast tumor | Not rs217727 polymorphism |
| 1. Maintenance of normal imprinting of H19 and IGF2 genes in neuroblastoma | Not rs217727 polymorphism |
